# A retrospective study of prostate cancer cases mimicking urothelial cell carcinoma of the bladder

**DOI:** 10.1186/2047-783X-18-36

**Published:** 2013-10-03

**Authors:** Ranlu Liu, Xiaoqiang Xie, Zhihong Zhang, Yong Xu

**Affiliations:** 1 Tianjin Institute of Urology & Department of Urology, Second Hospital of Tianjin Medical University, Tianjin 300211, China; 2 Department of Urology, the Second Hospital of Xiamen, Xiamen 361021, China

**Keywords:** Prostate cancer, Urinary bladder urothelial cell carcinoma, MRI, Differential diagnosis, Misdiagnosis

## Abstract

**Background:**

Prostate cancer (PCa) originating from the prostate base may intrude into the urinary bladder and may be misdiagnosed as bladder cancer. In this retrospective study, we reviewed the clinic data on PCa cases which were initially misdiagnosed as bladder cancer in order to identify diagnostic methods that would allow a better differential diagnosis for PCa.

**Methods:**

Out of a total of 455 patients treated for PCa at our hospital between April 2003 and June 2011, 14 patients (3.1%) had been initially misdiagnosed as urinary bladder urothelial cell carcinoma. The clinical data on these 14 cases was retrieved and analyzed.

**Results:**

Of the 14 patients, 11 patients were eventually diagnosed with PCa after MRI examination, and seven out of these had PCa with bladder neck invasion. Prostate needle biopsy or transurethral resection of prostate (TURP) revealed that all 14 patients had adenocarcinoma of prostate with Gleason scores ranging from 7 to 9. Nine patients received TURP for hematuria or lower urinary tract blockage. The mean follow-up was 37 months, during which six patients survived.

**Conclusions:**

As clinical presentation and in emergency settings, prostate cancer originating from the prostate base can be confused with bladder cancer originating from the neck or the triangle region of the urinary bladder. Serum prostate specific antigen (PSA) levels and digital rectal examination, in combination with transrectal ultrasound (TRUS), MRI, and prostate needle biopsy are valuable tools for definitive differential diagnosis of the basal prostate cancer.

## Background

The worldwide incidence of prostate cancer (PCa) is ranked second amongst all the cancers in men, with a mortality rate ranked sixth in the world. In many developed countries the incidence and mortality of PCa is even higher [1]. Although the incidence of PCa in China has been relatively low, in recent years it has shown an increasing trend, probably due to an increase in the aging population and changing life-styles. In general, at diagnosis, most of the patients are over 50 years old with mid-to-advanced stages of the disease. PCa affects the quality and expectancy of life, and is an increasingly important topic in the area of urology.

In terms of histopathology, McNeal *et al.* reported that 70% of the PCa tumors occur in the peripheral zone, 20% in the transitional zone and 10% in the central zone [2]. The central zone is located in the back of the prostate within the proximal urinary tract and displays an upside-down cone shape, where the cone bottom constitutes the base of prostate. PCa which originates from the base of prostate may enlarge and protrude into the bladder and form a regional hunch in the neck, trigone and posterior wall of the bladder. This is normally seen as intra-bladder-protruding lumps on the coronal plane during image examination and could be easily clinically misdiagnosed as a bladder-occupying lesion.

Thus, PCa tumor located at the prostate base and extending into the urinary bladder can be easily misdiagnosed as bladder cancer. Only a few studies have investigated criteria that would allow distinction between these two cancers [3-6]. The diagnostic tools used in these studies included urine cytology, immunohistochemistry and the use of molecular markers. In order to improve the clinical differential diagnosis of PCa from urinary bladder urothelial cell carcinoma, we reviewed and analyzed the clinical data of the misdiagnosed PCa cases. Clinical differential diagnosis using standard imaging, including transrectal ultrasound (TRUS) and CT and/or cystoscopy is not the same as pathological differential diagnosis. Therefore, this review mainly included the clinical findings, adjuvant diagnostic tests performed subsequently, the treatment given and the follow-up. The aim of this review was to identify the possible reasons that led to the misdiagnosis as well as to identify *clinical* diagnostic tools that allow distinction to be made between these two cancers.

## Methods

### Patients

Records of all PCa cases treated between April 2003 and June 2011 were reviewed to identify those that had been initially misdiagnosed as urinary bladder cancer. Data of *clinically* misdiagnosed cases which were later confirmed as PCa were analyzed. This study was approved by the Ethics Committee of the Institution in which it was performed. Written informed consent was obtained from every participant.

### Clinical diagnostic methods

Routine tests that had been performed on all patients were digital rectal examination (DRE), a blood test for prostate-specific antigen (PSA), prostate needle biopsy by the systemic 10-needle method, abdominal color Doppler, exfoliative urine cytology with acridine orange staining, and isotope emission computed tomography (ECT) bone scan. Other clinical diagnostics tests that had been performed on some of the patients included MRI, transrectal ultrasound (TRUS) examination, ^11^C-Acetate (^11^C-ACE) positron emission tomography-computer tomography (PET-CT), intravenous urography (IVU), cystoscopic examination, and microscopic biopsy.

## Results

### Patient characteristics

A total of 455 cases were treated for PCa during the eight year study period (2003 to 2011), and of these, 14 cases had been initially misdiagnosed as urinary bladder cancer. Detailed clinical data for these 14 patients was retrieved and analyzed. The age of the 14 patients ranged from 54 to 80 years, with a mean age of 70.6 years (Table 1). In terms of clinical symptoms, 12 of the 14 patients had reported frequent micturition and urgency of urination, eight had progressive dysuria due to bladder outlet obstruction, three had gross hematuria, and two patients reported intermittent painless gross hematuria (Table 1). Ten of the fourteen patients presented to the emergency department and four patients presented via the outpatient department.

**Table 1:** Patient characteristics

**Number**	**Age (years)**	**Clinical symptoms**	**PSA (ng/ml)**	**DRE**	**Cytology**^**2**^	**Gleason score**	**Clinical staging**	**Treatment**	**Follow-up duration (months)/outcome**
1	54	dysuria, frequent micturition (1 month)	11.5	III° enlargement, hard palpable lumps (right)	+	8	T4N1M1b	TURP surgical castration	22/death
2	72	frequent/urgent micturition (3 months)	19.9	II° enlargement, hard, no palpable lump	+	7	T4N1M1b	surgical castration	56/death
3	75	dysuria, frequent micturition (2 years) hematuria (1 month)	55.0	III° enlargement, soft, palpable lump (right)	-	8	T4N1M1b	TURP surgical castration	39/death
4	74	intermittent painless gross hematuria (6 years)	150.0	II° enlargement, hard, palpable lump (left)	-	7	T4N0M0	endocrine therapy chemotherapy	50/survival
5^1^	68	dysuria, frequent/urgent micturition (2 months)	31.7	III° enlargement, soft, no palpable lump	-	-	T4N0M0	TURP surgical castration	23/death
6	70	dysuria, frequent micturition (3 years) hematuria (1 week)	38.9	II° enlargement, hard, palpable lump (right)	+	7	T4N1M1b	surgical castration	40/death
7	58	dysuria, frequent micturition (1 month)	40.5	III° enlargement, hard, no palpable lump	-	7	T4N0M0	TURP surgical castration	30/survival
8	77	frequent/urgent micturition, (1 year)	30.5	III° enlargement, soft, no touchable lump	-	8	T4N1M1b	TURP surgical castration	loss to follow-up
9	69	dysuria frequent/painful micturition (1 year)	29.9	II° enlargement, hard, no palpable lump	-	7	T4N1M1b	surgical castration anti-androgen drug	47/survival
10	78	frequent/urgent micturition (1 year)	75.0	II° enlargement, soft, no touchable lump	-	7	T4N0M0	surgical castration	loss to follow-up
11^1^	80	dysuria, frequent urination (3 years), hematuria (4 days)	13.5	III° enlargement soft no palpable lump	-	-	T4N0M0	TURP endocrine therapy	21/survival
12	71	dysuria, frequent/urgent micturition (6 months)	27.0	III° enlargement, hard, palpable lump (right)	+	8	T4N0M0	TURP surgical castration	loss to follow-up
13	76	intermittent, painless, gross hematuria (1 month)	110.3	IV° enlargement, hard, no palpable lump	+	8	T4N0M0	TURP endocrine therapy	16/survival
14	66	dysuria, frequent/urgent micturition (6 months)	160.0	IV° enlargement, hard, no palpable lump	-	9	T4N0M0	TURP endocrine therapy	12/survival

DRE, digital rectal examination; PSA, prostate-specific antigen; TURP, transurethral resection of the prostate.

^1^patients diagnosed with HGPIN;

^2^exfoliative urine cytology analysis using acridine orange fluorescence staining has been done three times with the consistent results as shown as follows.

“°” means the degree of the enlargement of prostate.

Of the 14 cases, PSA levels were 10 to 30 ng/ml in four patients, 30 to 50 ng/ml in four patients, 50 to 100 ng/ml in three patients, and > 100 ng/ml in three patients, with an average value of 56.7 ng/ml (Table 1). The DRE revealed a hard prostate in nine patients, a soft prostate in five patients, second degree enlargement in five patients, third degree enlargement in seven patients, fourth degree enlargement in two patients, and palpable lumps in five patients (Table 1).

### Clinical adjuvant examinations

**Figure 1:**
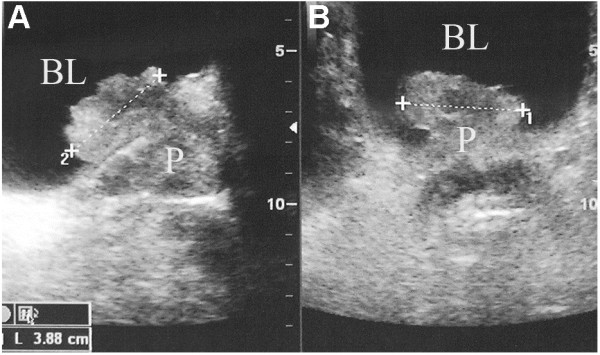
**Abdominal color Doppler ultrasound showing hypoechoic region in the bladder neck and trigone with mucosal thickening in the bladder neck, interpreted as a bladder-occupying lesion. (A)** sagittal plane; **(B)** transverse plane. P: prostate; BL: bladder.

All 14 patients had been examined in the outpatient or emergency department with abdominal color Doppler ultrasound of the urinary system and had shown a hypoechoic region within the bladder neck and trigone (Figure 1). Furthermore, seven patients had mucosal thickening in the bladder neck, trigone or posterior wall. Therefore, all 14 patients were clinically considered to have bladder-occupying lesions (Table 2).

**Table 2:** Interpretations of the diagnostic imaging

**Cases**	**Abdominal ultrasound diagnosis**	**CT scan**	**MRI**	**Bone ECT**	**Intravenous urography**
1	lesion in bladder neck	bladder lesion	PCa invading urinary bladder	multiple bone metastasis	normal
2	lesion in bladder neck	bladder lesion	PCa invading urinary bladder	multiple bone metastasis	normal
3	lesion in bladder trigone	bladder lesion	PCa invading urinary bladder	multiple bone metastasis	normal
4	lesion in bladder neck and trigone	bladder lesion	PCa	multiple bone metastasis	-
5	lesion in bladder neck	-	PCa invading urinary bladder	no metastasis	lesion in bladder
6	lesion in bladder neck	bladder lesion	PCa invading urinary bladder	multiple bone metastasis	normal
7	lesion in bladder neck and trigone	bladder lesion	PCa	no metastasis	-
8	lesion in bladder neck and trigone	-	PCa invading urinary bladder	multiple bone metastasis	-
9	lesion in bladder trigone	bladder lesion	-	multiple bone metastasis	normal
10	lesion in bladder trigone	bladder lesion	-	no metastasis	normal
11	lesion in bladder neck	bladder lesion (considered to be of prostate origin)	PCa invading urinary bladder	no metastasis	lesion in bladder
12	lesion in bladder neck and trigone	-	-	no metastasis	-
13	lesion in bladder neck	-	PCa invading urinary bladder	no metastasis	-
14	lesion in bladder neck	-	PCa invading urinary bladder	no metastasis	-

**Figure 2:**
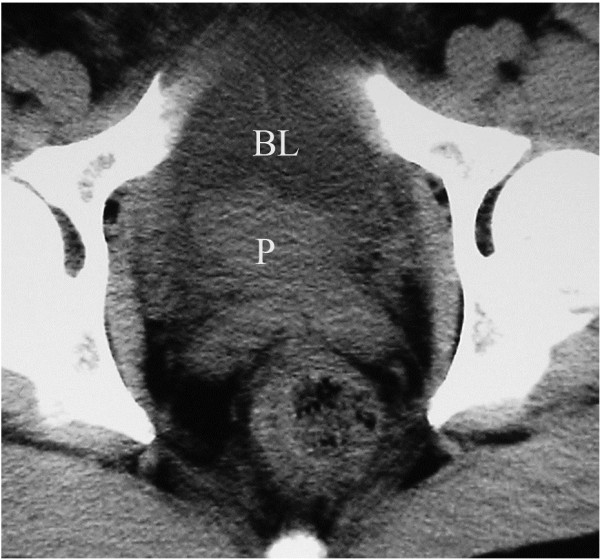
**CT indicates mucosal thickening in the bladder wall and a shadow of soft tissue density in the bladder neck, which was considered to be a bladder-occupying lesion.** P: prostate; BL: bladder.

The 14 cases showed varying degrees of prostate hypertrophy by ultrasound examination. The prostate size varied between 27.9 and 118.6 ml. Nine patients received a routine CT examination that showed an irregular soft tissue intruding into the bladder (Figure 2). Out of these nine, five patients had bladder wall thickening, three showed a blurred image in the trigone area and seminal vesicle, and four displayed enlargement in the lymph nodes surrounding the iliac blood vessels. Thus, the CT results suggested a bladder-occupying lesion. One case was considered to have a prostate originated lesion (Table 2). TRUS was performed for seven patients. Three of them showed a solid structure which invaded into the bladder neck, two patients showed a thickening of mucosa in the right bladder wall, and two patients showed a reduced ultrasound echo in the posterior subcapsular region of the prostate. Thus, a total of five patients were considered to have bladder-occupying lesions while two were considered to have prostate-occupying lesions invading into the bladder neck (Table 2).

Exfoliative urine cytology examination by acridine orange staining was positive for five of the 14 patients (Table 1). A routine intravenous urography (IVU) applied to eight patients, showed a bladder filling defect in two of them, suggesting a bladder-occupying lesion (Table 2). A routine cystoscopic examination performed on seven of the patients had revealed protruding lumps on the bladder neck and posterior wall. In general, the lumps had nodular structures, smooth surfaces, and wide bases, and in one case the node started from the posterior lip of bladder neck and extended to the left ureteral orifice**.** Microscopic biopsies were taken in two patients, and one patient revealed an invasive low-grade differentiated adenocarcinoma, the other patient indicated transitional cell carcinoma. The isotope ECT bone scan showed multiple bone metastases in six patients (Table 2).

### Clinical differential diagnosis

**Figure 3:**
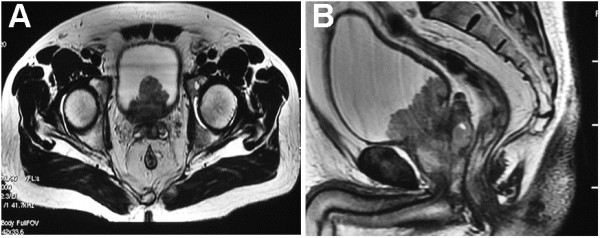
**MRI showing of one typical soft tissue.** MRI T2-weighted image showing an irregular abnormal signal of soft tissue shadow that intrudes into the bladder on the transverse plane **(A)**. The abnormal signal shadow intruding into bladder is a continuation of the prostate lesion on the sagittal plane **(B)**. The lesion was considered to be PCa with bladder neck involvement.

Of the 14 patients, 11 patients had undergone routine MRI examination. Obscure boundaries between the central and peripheral zones of the prostate were found in six patients. Low signal nodular shadow from T2-weighted images in the central or the peripheral zone was found in five and four patients, respectively. Also, irregular, abnormal signals of soft tissue shadow intruding into the bladder neck and posterior wall (Figure 3A), and continuation of bladder lesions to prostate lesions on the sagittal plane (Figure 3B) were found in nine patients. Blurred images were found in the trigone of the bladder and seminal vesicle in four patients. Finally, loss of normal structures in the prostate, with nodular protrusion, was found in five patients. Thus, on the basis of MRI, seven patients were considered to have PCa invading into the bladder, and four were considered to have PCa (Table 2).

Prostate needle biopsy (systemic 10-needle) performed on all 14 patients had indicated that 12 patients had PCa with a Gleason score ranging from 7 to 9 (Table 1). Two patients were diagnosed with high-grade prostatic intraepithelial neoplasia (HGPIN), but were later diagnosed as PCa by TURP with Gleason score of 7. Of the 14 cases, six cases were categorized as cT_4_N_1_M_1b_, and eight cases as cT_4_N_0_M_0_ (Table 1).

**Figure 4:**
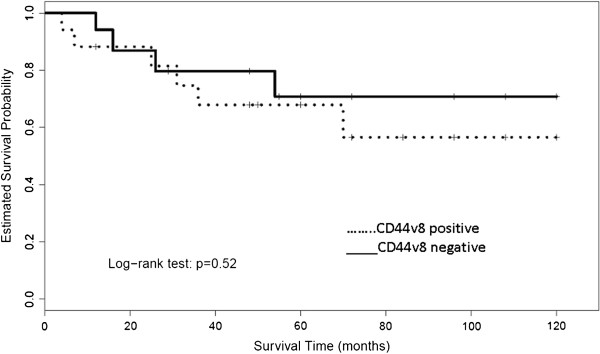
^**11**^**C-Acetate (**^**11**^**C-ACE) based PET-CT examination shows prostate enlargement intruding into the bladder with an abnormally increased metabolism.** Multiple lymph node enlargement **(A**, **C**, red arrow**)** accompanied by abnormally increased metabolism in the prostate and lymph node **(B**, **D)** is also shown. P: prostate; BL: bladder.

Two patients had undergone ^11^C-ACE PET-CT, which indicated an enlarged prostate intruding into the bladder with abnormally high metabolism, and multiple lymph node enlargements in the pelvic cavity with abnormally elevated metabolism (Figure 4).

### Treatment

All 14 patients were late stage high-risk patients who were not able to receive radical prostatectomy. Different strategies were used to treat the patients (Table 2): surgical castration in three patients, surgical castration plus TURP in six patients, surgical castration plus anti-androgen drug in one patient, TURP plus endocrine therapy in three patients (maximum androgen block, MAB) and endocrine therapy (MAB) plus chemotherapy (prednisone + docetaxel) in one patient. In nine patients who received TURP due to lower urinary tract blockage or hematuria, the Gleason score was revised from 8 to 9 in two patients, and in two cases, the diagnosis was changed from HGPIN to PCa. TURP findings were consistent with the biopsy results in the other five patients.

### Follow-up

Of the 14 patients, three were lost to follow-up and the remaining patients were monitored for 12 to 56 months, with an average follow-up duration of 32.4 months (Table 1). Of the six patients who survived till the end of the study period, two patients had well-controlled PSA levels without any apparent lower urinary tract symptoms, while four patients had uncontrolled high PSA levels and were diagnosed as castration-resistant prostate cancer (CRPC). These four CRPC patients were catheterized with a urinary catheter that was periodically replaced in case of dysuria. Of the 14 patients, five patients died due to multiple metastases, including bone, liver and brain metastasis.

## Discussion

In this retrospective study, we attempted to identify diagnostic techniques that proved to be useful in the *clinical* differential diagnosis of PCa to distinguish it from bladder cancer. Among the 455 PCa cases treated at our hospital, 14 (3%) had been misdiagnosed because the tumor was located in the base of the prostate and extended into the bladder. We discuss the clinical findings that aided in determining the correct differential diagnosis in these 14 cases which relied on PSA, MRI, microscopic biopsy, and ^11^C-ACE based PET-CT.

All of the 14 patients had received abdominal color Doppler ultrasound examination of the urinary system, which had revealed hypoechoic regions within the bladder neck and a protruded trigone extension into the bladder. CT scan in nine patients had revealed an intra-bladder, irregular soft tissue shadow. This was usually considered as a bladder-occupying lesion from the *clinical* point of view, though some prostate cancer could also protrude into the bladder and form a bladder-occupying lesion. When the PCa lesion is relatively large, the lesion itself or its upper-front prostate tissue may protrude into bladder. Furthermore, utilizing the axial CT scan results, it is sometimes hard to locate the lesion and the patients could be sometimes misdiagnosed as having bladder cancer. In this study, all of the misdiagnosis was based on the interpretation of abdominal color Doppler ultrasound and CT.

Prior to admission, these 14 patients were all *clinically* diagnosed as having bladder-occupying lesions and were examined with other adjuvant examination methodology based on the consideration of having a bladder-occupying lesion. In order to exclude upper urinary tract epithelial cancer, eight of the patients received IVU, which did not show any upper urinary tract filling defect.

Exfoliative urine cytology examination by acridine orange is a simple, non-invasive examination that is beneficial for the differential diagnosis of epithelial cell carcinoma originated from bladder and urinary tract [7]. However, the examination results may be affected by some subjective factors. Low-grade malignant tumor, trauma and inflammatory cells are hard to distinguish from one another and the false positive rate could be 15% to 30% [8]. At present, clinically performed exfoliative urine cytology examination still focuses on the observation of cell morphology, which does not include observation of surrounding tissue structures. In general, it is not easy to distinguish cancer cells and inflammatory cells by standard staining and observation under the light microscope. Urine samples of all 14 patients had been examined by acridine orange fluorescent staining of exfoliated cells and the results indicated five positive cases. From a certain point of view, this result could potentially mislead doctors to the diagnosis of urothelial cell carcinoma rather than PCa. Therefore, the utility of exfoliate cell examination is questionable for the differential diagnostic of PCa intruding into the bladder.

Krishnan and Truong [3] retrospectively studied 250 cases of high-grade carcinoma to identify clinical and urine cytological features that would allow differential diagnosis between prostate adenocarcinoma from transitional cell carcinoma. Eight of these cases (3%) were identified with PCa based on urine cytology at a frequency similar to the present study. A total of seven patients were checked by routine cystoscopy. Occupying lesions without typical papillary carcinoma findings in the bladder neck and trigone were observed. Microscopic biopsy was performed in two of the patients, one of which was diagnosed as a transitional cell carcinoma. Another case was diagnosed as invasive low-grade differentiated adenocarcinoma, possibly of prostate origin. Generally, when a tumor of prostate origin intrudes into bladder, the tumor is covered by the transitional epithelium. As the depth of biopsy is limited, the tissue taken is usually from bladder transitional epithelial mucosa above the tumor mass. Therefore, it is sometimes hard in these cases to obtain an accurate diagnosis by biopsy of a mass of prostatic origin, especially when the patient has inflammation, atypical hyperplasia or *in situ* carcinoma of the bladder trigone [9,10].

From the symptomatic view point, most of the patients in this study had lower urinary tract stimulation symptoms, while five patients had gross hematuria. These clinical manifestations are similar to those of bladder neck tumor infiltrating into deep muscular layer. Therefore, this can lead into a misdiagnosis of bladder cancer instead of prostate cancer.

At the international level, urologists commonly believe that a routine PSA and DRE examination should be carried out for males over 50 years of age with lower urinary tract symptoms. PSA level measurement and DRE play pivotal roles in the differential diagnosis of bladder cancer from prostate cancer [11]. All of the patients had various degrees of PSA elevation in this study. However, the serum PSA level may be influenced by catheterization, urinary retention or DRE. Meanwhile, high-grade (Gleason score 8 to 10) prostate cancer may have low (< 10 ng/ml ) or even normal (< 3 ng/ml) serum PSA. The clinical differential diagnosis became more difficult in this situation. Therefore, urologists should consider the possible influence of false positive PSA results in these situations. DRE was performed in ten patients with five of them showing palpable lumps, which was an indication of PCa.

Among the imaging techniques, MRI appears to be more useful in the diagnosis of PCa than CT scan. It can provide multi-dimensional images and produce high resolution axial images of both sides of the peripheral lobe and the surrounding lesion. The lesions on the bottom and tip of prostate are easily observed on the coronal and sagittal plane and T2-weighted MRI images [12]. A total of 11 patients in this group were routinely examined by MRI. In nine patients, an irregular abnormal soft tissue shadow protruded into the bladder and connected with the lesion in the prostate on the sagittal plane. Therefore, they were diagnosed as PCa based on MRI. Also, seven patients were diagnosed as having PCa with bladder involvement. The other four patients were diagnosed as PCa. The results were consistent with the final pathological diagnosis. However, MRI is expensive, operator dependent, and time consuming with some limitations, especially in the emergency setting. Therefore, we suggest that TRUS performed by an expert operator is the first approach to study the prostate anatomy. Based on TRUS findings, further imaging is required or prostate biopsy is performed.

PET-CT has a very important role in localization and distinguishing of the malignant tumor and its metastases [13]. Two of the patients were examined with ^11^C-ACE-based PET-CT scan, which further confirmed that the lesions originated from the prostate and had pelvic lymph node metastasis. However, PET-CT is expensive and cannot replace needle biopsy, and is therefore not an essential item for diagnosis.

Prostate needle biopsy is the most reliable examination for the diagnosis of PCa [14]. In this study, because all of the 14 patients had high levels of PSA, they were checked by transrectal prostate needle biopsy using the systemic 10 needle method. The results indicated PCa in 12 of them, and HGPIN in two of them. Furthermore, TURP was performed in the latter two patients because of dysuria, which indicated PCa. The reason for misdiagnosis of a biopsy may be that the tumor originated from the central lobe and protruded into the bladder and so the routine transrectal needle biopsy cannot reach the tumor position. Therefore, transperineal template-guided saturation biopsy should be considered when the suspect tumor is in the central zone or anterior region [15].

McNeal *et al.*[16] have reported that PCa originating from the transitional region is usually diagnosed by TURP examination. In this study, we focused on PCa originating from the central lobe and undertook examination with TURP to avoid misdiagnosis. All of the 14 patients were late-stage and high-risk patients and were primarily treated with endocrine therapy. Furthermore, nine patients were treated with TURP for hematuria or lower urinary tract blockage. Eleven patients were regularly checked during the follow-up. Six patients survived during the follow-up period; however, four patients progressed to CRPC. These findings demonstrated that PCa originating from the central lobe has tendencies to bladder neck infiltration and remote metastasis. Therefore, it has a low curative potential and a relatively bad prognosis.

## Conclusions

Taken together, the data presented here suggest that when lesions are found in the neck, trigone or posterior wall of the urinary bladder, the possibility of basal prostate cancer should be considered. For the middle aged and elderly male patients with lower tract blockage, bladder stimulation, bone pain, hematuria and other symptoms, routine PSA and DRE examinations should be performed. TRUS or MRI examination is suggested when necessary. During imaging, it is important to look for a prostate with an enlarged basal region protruding into the bladder, and a prostate with a disorganized structure. Furthermore, it is important to examine whether the symmetry is disrupted, whether the capsule is intact. A multi-view analysis can clinically reduce the rate of PCa misdiagnosis. Finally, prostate biopsy should also be performed when prostate cancer is suspected.

## Abbreviations

PCa: Prostate cancer; TURP: Transurethral resection of prostate; TRUS: Transrectal ultrasound; PSA: Prostate specific antigen; DRE: Digital rectal examination ECT, emission computed tomography; PET-CT: Positron emission tomography-computer tomography; IVU: Intravenous urography; HGPIN: High-grade prostatic intraepithelial neoplasia; MAB: Maximum androgen block; CRPC: Castration-resistant prostate cancer.

## Competing interests

The authors declare that they have no competing interests.

## Authors’ contributions

RL and YX conceived and designed the clinical study, RL drafted the manuscript, XX and ZZ carried out the study. All authors read and approved the final manuscript.
